# The experiences of counselors caring for children and adolescents with problematic smartphone use

**DOI:** 10.1038/s41598-023-39494-8

**Published:** 2023-07-29

**Authors:** Jaewon Joung, Eunhee Oh, Eun Jee Lee

**Affiliations:** 1 College of Nursing, Research Institute of Nursing Science, Jeonbuk National University, 567 Baekje-daero, Deokjin-gu, Jeonju-si, 54896 Jeollabuk-do Korea; 2 Department of Nursing, Hyejeon College, Hongseong, Korea

**Keywords:** Health care, Medical research

## Abstract

This study examines field experts’ experiences to ascertain the actual circumstances and strategies to increase the efficacy of intervention programs for children and adolescents with problematic smartphone use. Three focus group interviews were conducted via video conferencing. The data were grouped into three major themes: (1) the screening and inflow phase, which included the inaccuracy of the screening tests, barriers in the inflow process, and the importance of school cooperation; (2) the intervention phase, which included the necessity of developing a program tailored to the target audience, the importance of parental participation, and concerns about the vulnerable; and (3) the maintenance phase, which included the lack of a long-term strategy, the need to re-establish the purpose of the counseling/intervention programs, and the need for systematic maintenance of the system. To improve the reliability of the screening test for children and adolescents with problematic smartphone use, it is necessary to improve the measurement tools and environment. To increase parental involvement, education should be provided on perceiving the severity of problematic smartphone use, and program running hours should be varied. The findings offer information necessary for improving counseling and interventions for children and adolescents with problematic smartphone use.

## Introduction

In this rapidly changing world, children and adolescents learn communication and new information through technological devices such as smartphones and tablet PCs^1^. The coronavirus disease 2019 (COVID-19) pandemic has accelerated digital globalization, further increasing the use of technological devices^2^.

The COVID-19 pandemic had a profound impact on smartphone use among children and teens. Problematic smartphone use (PSU) rates in children and adolescents are the highest among all age groups. During the COVID-19 pandemic, the risk of PSU in children and adolescents significantly increased from 30.2 to 37.0% in 2021^3^. In particular, smartphone and Internet usage time increased by 73.8% compared with that before the COVID-19 pandemic^4^. Using smartphones for too much time can lead to PSU.

During childhood and adolescence, when growth and development are rapid, PSU has adverse effect. PSU during this period not only has adverse effects on adolescents whose brain development is incomplete^5^ but also negatively affects eyesight^6^ and causes sleep disturbance and obesity^7^. In addition, it affects mental health and the ability to control negative emotions such as self-blame^8^, which may cause conflicts with family and friends, and is associated with suicide attempts^9^. PSU can cause difficulties in adjusting to school life in children and adolescents^10^, and reduced opportunities for interpersonal interaction with peers during the critical youth period to develop as competent members of society can negatively affect the formation of sociality^11,12^. In other words, PSU causes a variety of physical, mental, and social problems for children and adolescents.

In South Korea, PSU among children and adolescents is receiving national attention. The Korean government established the Third Comprehensive Plan for Prevention and Relief of Internet Addiction. In addition, annual national surveys have been conducted, and infrastructure and a treatment cooperation hospital system have been established^13^. In South Korea, a screening test for PSU is administered annually to all students in the fourth grade of elementary school and the first year of middle school (seventh grade). If a student is found to be at high risk of PSU, their records are transferred to professional institutions (Korea Youth Counseling & Welfare Institute), which then contacts the parents to provide counseling and interventions. The services children and adolescents with PSU receive include individual counseling, group therapy, and parent–child interventions, all of which are developed by the government and provided to institutes. Despite this program, PSU in children and adolescents has continued to worsen during the COVID-19 pandemic^3^.

As the number of children and adolescents with PSU increases, research in the area is increasing. As youth have the highest PSU rate among all age groups, most previous studies have focused on this group^14–17^. However, the age of first smartphone has gradually decreased, and the elementary school children are the new target for preventing PSU^18^. Furthermore, it is difficult to determine the lasting effects of PSU intervention programs for children and adolescents^19^, and there is a lack of such programs involving parents or teachers.

Therefore, it is necessary to fill the gap in the current research and to provide a basis for countries to develop policies for appropriate use of smartphones by children and adolescents. Focus group interviews (FGIs) enable people in the same field of work to participate in discussions and comfortably disclose their experiences, feelings, and beliefs through intragroup interactions^20^. Therefore, we conducted FGIs with counselors who are experts in PSU counseling and intervention programs for children and adolescents. This study aimed to investigate policy and practical problems as well as interventions for PSU in children and adolescents. The results of this study provide insights for improving PSU counseling and intervention programs for children and adolescents in Korea.

## Methods

### Study design

This qualitative study employed focus group interviews to understand the PSU counseling experts’ experience with children and adolescents and to seek ways to improve the effectiveness of PSU treatment programs.

### Participants

The participants were experts from institutions that administer PSU counseling and intervention programs for children and adolescents, such as the Korea Youth Counseling and Welfare Institute, Local Community Addiction Management Center, Wee Center (Centers that provide guidance for children and adolescents in cooperation with schools, the Offices of Education, and the local communities), psychiatric hospitals, and private psychological counseling clinics. As active and in-depth discussions can be ensured when FGI participants’ work backgrounds are fundamentally similar^21^, groups were assigned according to institution. The detailed criteria for the selection of study participants are as follows:

1. Those who had directly conducted intervention programs or counseling for children and adolescents with PSU for more than a year.
2. Those who understood the study’s purpose and method and voluntarily agreed to participate

### Data collection

The FGIs were conducted from January to March 2022. The interviews were conducted three times, once in each of the three groups, and the number of participants in each group was 3, 3, and 6 (12 participants in total) (Table 1). Online interviews were conducted after confirming that all participants were proficient in video conferencing owing to the COVID-19 pandemic. Interview questions were shared a day before. The interviews were conducted for 90 min for each group. The main interviewer was a researcher with extensive experience in FGIs; the other researchers noted important content and non-verbal responses. Five types of interview questions were derived based on the practical guide for FGI^20^ and literature reviews, and the final questions were determined after discussion by the three co-researchers (Table 2).

**Table 1:** Participants’ general characteristics.

ID	Gender	Age	Work experience (years)	Main work (multiple)	Title
A	Woman	35	12	Personal counseling	Team member
B	Woman	52	20	Personal counseling	Center director
C	Woman	38	18	PSU intervention program	Team member
D	Woman	41	4	Personal and group counseling	Team member
E	Woman	48	5	Personal counseling	Counselor
F	Man	31	4	Personal counseling, PSU intervention program	Team member
G	Woman	47	2	Personal counseling, PSU intervention program	Team member
H	Man	31	4	Personal counseling	Team member
I	Man	40	8	Personal counseling	Team leader
J	Woman	32	9	Personal counseling	Team member
K	Woman	44	15	Personal counseling, PSU intervention program	Team leader
L	Woman	28	2	Personal counseling, PSU intervention program	Team member

*PSU* problematic smartphone use.

**Table 2:** Interview questions.

Category	Questions
Opening question	Please introduce yourself, including your current work
Introductory question	Please share your experience in intervention programs or counseling for PSU
Transition questions	What are the main characteristics of parents who request participation in PSU counseling or programs? Were there any particularly memorable parents? Please tell us about your experience
Key questions	Main question How was the experience of conducting counseling/intervention programs for school-age children and adolescents with PSU? How was the counseling/program operated for school-age children and adolescents with PSU? What were the good points of the counseling/program for school-age children and adolescents with PSU? What should be improved about the counseling/program for school-age children and adolescents with PSU? Supplementary questions What are the characteristics of parents who exacerbate children's PSU? What are the characteristics of parents who relieve children's PSU?
Ending questions	What are the most important things that we have discussed today? Is there something important that we did not discuss?

*PSU* problematic smartphone use.

The recordings were transcribed into text files using a speech-to-text program, and a research assistant corrected the transcriptions to ensure they matched the actual data. A total of 71 pages of transcribed interview files and field notes written by two researchers was used for data analysis. Transcriptions were cross-checked between researchers to ensure accuracy.

### Data analysis

The FGI data were analyzed using the thematic analysis method of Braun and Clarke^22^. This method is used to describe patterns or meanings and to identify a topic. The specific analysis methods were as follows:

1. Read and understood the transcriptions repeatedly to understand the data as a whole.
2. Grouped similar content and assigned initial codes, within which content again was grouped by similarity.
3. Grouped codes together to generate potential themes.
4. Reviewed the relationships between the topics and the coded data, iterating on similar topics and categorizing them into higher-level topics.
5. Clearly distinguished, named, and defined the meaning of each topic.
6. Extracted examples to illustrate the themes and wrote a report.
7. Two researchers conducted the primary analysis, contrasted the outcomes, and resolved inconsistencies either using a third reviewer or through consensus.

### Rigors

The researchers attempted to satisfy the four criteria of validity of Sandelowski^23^: credibility, fittingness, auditability, and confirmability. First, to ensure the credibility of the study, we selected experts from institutions that operate PSU counseling or intervention programs as the research participants, excluding personal acquaintances. The interviews were held in the evening so that the participants could participate in a comfortable and private place after work. The researchers asked open-ended questions so that the participants could express their opinions freely. In addition, the participants' interview content was transcribed within 24 h of the interviews to minimize omissions and distortions. The researcher triangulation method was used to derive credible results. Researchers had continuous discussions until an agreement was reached, ensuring personal judgements and biases did not affect the interview and outcome reporting. Second, in terms of ensuring fittingness, participants were carefully selected to avoid bias related to age, work experience, work institution, and work position to minimize elite bias (Table 1). Interview content was confirmed by participants at each interview by providing interview summaries to ensure that the researchers understood the interview content correctly. The interviews were conducted until data saturation was achieved. Third, in terms of ensuring auditability, the research purpose, data collection methods, analysis process, and interpretation are described in detail to help readers understand. Finally, in terms of ensuring confirmability, the authors attempted to minimize bias and prevent the transmission of the researcher's feelings and experiences to the participants.

### Ethical considerations

This study was approved by the Institutional Review Board (IRB) of the Jeonbuk National University (Approval no. JBNU 2021-11-001-003). All methods were performed in accordance with the relevant guidelines and regulations. This study only included participants who voluntarily agreed to participate. The researchers fully explained the purpose and methods of the study to the participants prior to the interviews. Participants were informed that they could withdraw their participation at any time. Additionally, the participants were aware that anonymity would be secured and that the data collected would only be used for research purposes. Prior to the FGIs, the participants provided their informed consent to record the interviews. The content of the interviews was not criticized and was kept confidential. All the members participating in the interviews, including the participants and research assistants, signed a non-disclosure agreement.

## Results

The participants expressed their perspectives on children and adolescents with PSU. A detailed summary of the findings is presented in Table 3.

**Table 3:** A summary of the themes identified in the study (N = 12).

Category	Subcategory	Contents
1. Screening and inflow process	Inaccuracy of screening test	The need for improving testing environment
		Increasing reliability of screening test
	Barriers in the inflow process	Association of various psychosocial problems
		Lack of parental perception of severity of PSU
		Social stigma
	The importance of school cooperation	Uncooperative school
		Cooperative school
2. Intervention	Programs requiring supplementation	Utilizing various media
		Emotional support rather than cognitive approach
		Focusing on alternative activities rather than behavioral control
		Considering sociodemographic backgrounds
	The importance of parental involvement	Improving parent–child relationships
		Emotional intervention needed for parents
		The need for identifying ways to increase parental participation
	Concerns about COVID-19 and vulnerable groups	Increase in smartphone use due to online learning
		Special measures required for the vulnerable
3. Follow-up care	Lack of long-term strategies	Importance of follow-up care
		Requiring case management services
	Need to re-establishing program goals	Focusing on adequate use rather than control
		The necessity of setting clear goals
	Need for system reorganization	Considering the time and method of providing various services
		The necessity of integrating duplicate services

*PSU* Problematic smartphone use, *COVID-19* Coronavirus disease of 2019.

### Screening and inflow process

The experiences of experts in selecting and recommending children and adolescents with PSU for counseling were as follows:

#### Inaccuracy of screening test

To accurately screen children and adolescents with PSU, a relaxing environment with guaranteed privacy and detailed explanations are needed. However, sometimes, test explanations were provided in open school classes with other peers. Consequently, they completed the tests without proper understanding, which led to random and insincere answers that subsequently reduced the reliability of the results. In such cases, the test could potentially under- or over-estimate PSU during the screening.

When screening tests were completed in groups or public places, the children did not provide honest answers. In relaxed and private spaces for one-on-one conversations where the children could open their minds, they seemed to better understand the explanations, which increased the reliability of the test results (J).

Additionally, the results of the PSU screening test were predictable. Older adolescents seemingly provided dishonest answers to avoid being classified as suffering from PSU. Therefore, a single screening test is limited in identifying children and adolescents with PSU. Additional tests, such as emotional and behavioral tests, are required.

The test questions are predictable, and the children can provide dishonest answers. They know how to obtain high test scores. Instead of providing honest answers, they provide insincere answers to avoid falling in the overdependence category (K).

#### Barriers in the inflow process

Children and adolescents with PSU also have complex problems such as depression, inability to control impulses, learning disabilities, behavioral problems, and vulnerable families. Therefore, the participants needed to construct programs that considered various psychosocial problems, in addition to PSU.

One child had a lot of problems with smartphones. After the child was referred to our center, our test results showed that the child also had an inability to control impulses, poor learning ability, and difficulties in concentrating (C).

In other cases, parents had a poor understanding of the seriousness of their children's conditions. In these cases, the parents did not cooperate, expressed discomfort, and were against seeking help to treat their children’s PSU.

The parents mostly expressed discomfort about why their children had to visit addiction centers and undergo counseling for PSU. This presented difficulties regarding consultations (J).

Concerns about mental health-related stigma prevalent in Korean society also contributed to parents’ negative perceptions and hindered adequate help in treating PSU in children and adolescents.

The parents refused to allow their children to receive counseling for concerns such as “the counseling will be on the records’ and strongly opposed leaving any records (J).

#### The importance of school cooperation

School cooperation plays an essential role in the process of encouraging the participation of children and adolescents with PSU. Schools that actively cooperate in resolving PSU problems have been of great help in introducing treatment programs for children and adolescents. However, schools’ awareness of the importance of PSU management was insufficient, and there were some cases in which cooperation regarding time and space for intervention was not well achieved.

The results varied depending on the school's cooperation. The school is the first to identify this problem. Problematic behavior is often more pronounced in peer relationships (A). The teachers, especially principals and vice principals, were not cooperative. They did not allow us to talk to the students during class, and we could not find an appropriate time for counseling. I think there is a need for changes in teachers’ thoughts and mindset (D).

### Intervention

The experiences of the participants in providing interventions to children and adolescents with PSU were as follows.

#### Programs requiring supplementation

In Korea, a national survey on PSU in children and adolescents is conducted once a year, and individuals who are categorized into the PSU group are provided counseling and group programs by youth counseling-related organizations based on manuals developed by the Korea Youth Counseling & Welfare Institute. However, the basic manuals do not attract the interest of children and adolescents. Thus, the participants invested their efforts in drawing the interest of children and adolescents and communicating with them by developing various contents.

We use the program provided by the Korea Youth Counseling & Welfare Institute, but add extra content that children and adolescents will find interesting. For example, media such as videos and quizzes using messenger apps help provoke children's interest (K). Children experience emotional difficulties that cannot be expressed through words. Playing games with them, rather than communicating verbally, helps to develop intimacy. In doing so, children present more of themselves, feel a sense of accomplishment, and communicate with us. That is why we always use media such as art and board games (B).

Moreover, the experts agreed that an emotional approach was more important than a cognitive approach, such as education, when constructing interventions. In particular, understanding psychosocial problems masked by PSU and providing emotional support had positive effects on the interventions.

It seems that children with psychosocial difficulties tend to become more dependent on their smartphones… Some children become more obsessed with social media, while some children become more addicted to games to have their sense of satisfaction... (omitted) Understanding their thoughts helps build relationships with them. Subsequently, the children tend to express themselves more. After building relationships, I introduced the program and proceeded with counseling without any problems (A).

Another effective strategy for children and adolescents with PSU was to seek alternative activities rather than behavioral control. As children and adolescents were appreciated by others through alternative activities, they gained confidence and showed a reduced desire to use smartphones, which occasionally led to a turning point in their lives.

I often suggest alternative activities to replace smartphone use. Let's find more fun activities instead of using smartphones! For girls, I mostly suggest crafting; for boys, I recommend exercises (L).

In constructing appropriate interventions, it is important to consider differences in sociodemographic backgrounds, such as the children’s age, gender, and academic level. The participants were aware that implementing the intervention according to groups was more effective.

I think it will be different for each age group. Elementary school students can stay with their parents overnight, but middle and high school students have a hard time staying that long with their parents (H).

#### The importance of parental involvement

When parents, who were previously uninterested in their children and had a dysfunctional relationship with them, improved their relationship with their children through interventions, PSU tended to improve as well. Therefore, the experts stated that promoting parental participation in the intervention was desirable.

Children who do not have a good relationship with their parents tend to become more addicted to smartphones. At first, they sit separately from their parents. However, at the end of the program, I see the parents and children closely together. The child leans on the parent, sits on the lap, or holds their hands. I definitely saw changes in the follow-up tests for such children (K).

On the other hand, participants expressed that parents who have suffered from their children's problems also require active emotional support and need to be looked after.

Many people say to parents, "understand children,” "respect children,” but I think parents also need to be respected or supported emotionally. It is important to also cater to the emotional needs of the parents, and we need more effort to help parents understand their children in an environment where they can be understood as well (J).

Therefore, the participants sought different ways to increase parental participation and overcome various obstacles such as lack of awareness and limited free time for intervention to treat PSU in children and adolescents.

The real problem cannot be solved if parents cannot be seen. Perhaps it would be ideal to conduct the program, once a week or at least once a month, even after parents' work hours (F).

#### Concerns about COVID-19 and vulnerable groups

Online learning continued for more than 2 years as schools were closed for social distancing purposes during the COVID-19 pandemic that began in early 2020. Consequently, children and adolescents inevitably spent more time on their smartphones, which increased their PSU.

Children have been using smartphones much more since the COVID-19 pandemic started. Most cases of PSU occur when children cannot control their smartphone use while engaged in remote online classes. Last year's survey also showed that children are using smartphones more often than before COVID-19 pandemic (L).

Children and adolescents living in rural areas, multicultural families, single-parent families, and low-income families tend to have more serious PSU. The participants expressed that, as families of such children and adolescents have difficulties managing their usual lifestyle and participating in interventions, special national measures for children and adolescents in vulnerable groups need to be provided.

Children living in urban areas have various activities to share with their friends, but children in rural areas have nothing to do except use smartphones. So, I cannot dare tell them not to use their smartphones. All adults work on farms, and they do not have friends of similar age. When they go downtown, many people have mental disorders, which can be dangerous for children. Sometimes, it is safest for them to play with their smartphones at home (H).

### Follow-up care

Participants who had experience providing interventions for children and adolescents with PSU suggested the following problems and future strategies for interventions.

#### Lack of long-term strategies

After short-term intervention, the children and adolescents showed positive improvements. However, this problem recurred over time, suggesting the importance of follow-up care.

Group programs were provided once a week for only five weeks. People cannot change in just five weeks. I think that counseling sessions should be provided regularly every month or two weeks for a long period after the five initial sessions (E).

Case management services were suggested for follow-up management. Participants expressed that effective intervention without recurrence would be achieved through long-term rather than one-time interventions with formality.

I think that case management services are needed instead of program services. I thought that case management would help provide more long-term interventions (F).

#### Need to re-establish program goals

Since the COVID-19 pandemic, computers and smartphones have become key devices in the lives of children and adolescents. Therefore, the participants also suggested that awareness and education on adequate use are necessary rather than preventing use.

I wonder, if we cannot stop using smartphones, how about educating children on how to use them in a healthy way? Various useful pieces of information can be obtained from YouTube. We should teach children and adolescents the right and healthy way to use smartphones (B).

Although the participants devoted much effort, there was still a lack of confidence in the standard criteria for intervention. The participants indicated that various discussions were needed to establish clear target standards for effective interventions. In addition, the participants believed that the criteria for evaluating goal achievement needed to be defined more clearly.

I do not know exactly about its effectiveness. It is unclear whether the reduced time of smartphone use is effective or whether children will develop self-control (E).

There are limits to quantitatively expressing evaluation methods in numbers. I think qualitative evaluations must also be conducted. (F).

#### Need for system reorganization

Various approaches are needed regarding the method and time of intervention provided to parents, as well as to their children and adolescents. For example, interventions should be provided after work hours or on weekends for parental participation. If face-to-face education is difficult, interventions should be provided in a contactless manner through an online video conferencing system, and adequate measures must be sought to increase the effectiveness of interventions. Furthermore, interventions involving parental participation must include detailed considerations and meticulous strategies, such as potential feelings of pain or alienation that children and adolescents who do not live with their parents may feel.

PSU is closely related to children’s relationship with their parents, but some children do not live with their parents. I suddenly thought about how those children might feel if they participated in a program that required parental participation (D).

Another concern is that multiple institutions provide similar interventions. A clear consensus on children and adolescent addiction and emotional behavioral problems needs to be established by an organized body to avoid duplicate interventions.

There are competitions for similar interventions. I hope we can have a unified system to avoid overlapping service interventions and to establish a systemic plan for children (J).

## Discussion

This study aimed to explore the experiences of counselors who care for children and adolescents with PSU. The results of this study are as follows.

First, in FGIs, participants suggested potential problems with tests to screen for children with PSU. The currently available screening test tool for PSU consist of items with predictable results. This can lead to inaccurate and dishonest answers, which may reduce the reliability of the screening test. The PSU tool used in previous studies closely resembles a simplified scale and cannot be generalized. Thus, this tool is mostly used for formality^24^. Moreover, as many children and adolescents with PSU also have mental health problems, such as depression, anxiety, loneliness, impulsive behavior, and high stress levels^25–27^, tools with greater reliability are needed to accurately screen for children and adolescents with PSU, and emotional and behavioral tests must be conducted simultaneously for comprehensive analysis.

PSU intervention programs for children and adolescents must be structured for flexible application under various circumstances. The current standard program provided by national institutions needs to be supplemented. As previously indicated, smartphones have become an escape route for children and adolescents with psychosocial problems to avoid negative emotions. Our findings show that it is important to provide compassionate care that sensitively examines and supports the emotional factors of children, in addition to adopting a cognitive approach, which is consistent with the findings of previous studies^25–28^. Additionally, our participants reported that standard interventions must include a variety of content for interest arousal and the developmental stage of children. This is consistent with previous findings, in which experts added solution-focused short-term therapy, existential therapy, motivational interviewing, art therapy, and career counseling based on physical education, music and art activities, and the growth stages of children and adolescents to arouse their interest^24,29^. The participants also highlighted the importance of alternative activities, which can reduce interest in smartphones and promote a sense of accomplishment, self-control, and self-esteem in smartphone-overdependent children, who are often helpless and have low self-esteem^29,30^. Therefore, in addition to the PSU standard program, tailored content targeting each individual, motivational activities, correct coping strategies, compassionate care, and alternative activities must be created for flexible application to different groups of children.

In our FGIs, the participants also expressed the importance of programs for parents with children who are overdependent on smartphones. Many parents were unaware of the seriousness of their children's PSU and were reluctant to allow them to participate in intervention programs because of fear of stigma. The parents were passive in participating in the programs or counseling sessions due to their fear of being labeled as parents with a problematic child and the shame of having their family or parenting problems out in the open^31^. In such cases, PSU may worsen, as children and adolescents miss the ideal window for intervention. Thus, parents must improve their awareness of the seriousness of the problem and acknowledge that children require attention and support rather than stigma while having higher expectations for professional help. Additionally, the experts described the importance of parental participation for an effective intervention. As reported in previous studies, high trust and stable attachment between children and parents, formed through proper parenting, enhance self-regulation in children and adolescents^32–34^. When parents are overly controlling or fail to act as parents, their children use smartphones more often^24^. Therefore, to control PSU in children and adolescents, it is important to improve parent–child relationships, restore family functions, and increase parenting efficacy and positive parenting capacity. Parental mediation programs that include education on the proper control of smartphone use while inducing positive relationships with children need to be prepared. Therefore, it is necessary to ensure privacy and confidentiality for parents and children to participate in PSU programs, and to improve public awareness of overdependence intervention programs through mass media and parent education in schools. School cooperation is another important factor in reducing PSU among children and adolescents. The lack of cooperation by schools has led to difficulties in accurately selecting children and adolescents with PSU and providing adequate intervention due to temporal and spatial restrictions. This suggests that our society, including schools, is unaware that PSU in children and adolescents is an important issue that needs to be resolved urgently. Consistent with our findings, previous studies have demonstrated that schools and teachers must cooperate to develop and evaluate programs with active support from the Ministry of Education^29^. As children and adolescents spend most of their time in school, cooperation among schools is essential to effectively prevent and manage health problems.

After the COVID-19 pandemic, the world has become rapidly digitized, and children and adolescents spend more time on their smartphones as they attend online classes^2,35^. Owing to the quarantine and social distancing measures, smartphones have become the most convenient devices for children and adolescents and act as the most important channel for information and interaction with society^25^. However, parents are reluctant to tolerate their children's smartphone use^2^. This greatly increases the PSU rate^3^, and the participants in our study experienced more cases of PSU and counseling. In particular, children and adolescents of vulnerable groups, such as those in rural areas, multicultural families, single-parent families, and low-income families, have serious PSU, which only worsened during the COVID-19 pandemic. Parents work long hours to make a living and do not have enough time to monitor their children's smartphone use^25,35^. Consequently, children may not have sufficient opportunities for psychosocial development and may be deprived of future opportunities^27^. Therefore, there is an urgent need to prepare support measures to address the effects of the COVID-19 pandemic among vulnerable groups of children and adolescents. In addition, education on the appropriate use of digital devices and safety literacy improvement must be developed and applied while seeking alternative activities to replace smartphone use in vulnerable groups of children and adolescents.

Several problems were observed during follow-up care. Our findings show that an integrated system for PSU management must be developed and managed through cooperation with local communities and agencies. Currently, similar services are provided by different organizations. The participants expressed concerns that there are unclear standards regarding whether the goal of counseling and intervention is to reduce smartphone use or to develop children's self-control. Therefore, schools, parents, experts, and government agencies must reach consensus on the ultimate goal of PSU intervention. Finally, follow-up measures are needed for continuous management after the completion of the interventions. Compared with the seriousness and importance of PSU in children and adolescents, there is a lack of proper follow-up measures after the intervention. This is consistent with previous findings that reported short-term counseling^24^ and a lack of transition from group programs to individual counseling^29^. Countries need to stabilize sufficient financial resources and integrate and systematically manage duplicate services. Systemic changes can produce resistance from constituents, which might require collection of constituent input, establishment of communication channels, and appropriate training opportunities for new professionals^36^.

In this study, we investigated the current situation and potential improvements in PSU-related interventions through the experiences of counseling experts. This study is meaningful as it provides basic knowledge for a more effective smartphone dependence management system following the gradual increase in PSU. However, this study is based on interviews with professionals who counsel children and adolescents with PSU in Korea, so it is necessary to be cautious about generalizing the experiences to all professionals with diverse cultural and social backgrounds. In future studies, it is necessary to conduct interviews with professionals from various cultures and to collect data from various sources such as observations, documents, and surveys to gain a comprehensive understanding of PSU management. Also, exploring additional factors that influence intervention programs and evaluating long-term outcomes, considering different cultural contexts, will help to advance the field of practice. Last, this study only reported the opinions of counseling experts. Therefore, assessment of the experience of children and adolescents, parents who are directly involved in interventions, and schools and teachers who first identify the problem and refer cases to relevant services should also be considered in future studies.

## Conclusions

FGIs were conducted to assess the status and potential improvements in PSU treatment programs for children and adolescents. Our findings have the following implications. The reliability of the screening test needs to be increased, and intervention programs must be supplemented with various contents and media. Parents’ and schools' awareness of PSU needs to be improved, while schools must actively cooperate to provide adequate interventions for children and adolescents. Additional programs for low-income families should be developed, and a systematic approach must be adopted, especially by an organized body. The implications of this study are as follows. First, a systematic intervention program development and effectiveness verification study must be conducted to address the problems identified in this study. Second, it is necessary to develop mental health policies at a social level to increase public awareness of the seriousness of PSU during the COVID-19 pandemic.

## Data Availability

The datasets generated during and/or analysed during the current study are available from the corresponding author on reasonable request.
